# Three Synchronous Pituitary Neuroendocrine Tumors—Epigenomics Confirm an Exceptional Triple PitNET

**DOI:** 10.1007/s12022-025-09864-1

**Published:** 2025-05-16

**Authors:** Temor Rafiq, Jakob Matschke, Jörg Flitsch, Matthias Dottermusch

**Affiliations:** 1 Institute of Neuropathology, University Medical Center Hamburg-Eppendorf, Martinistr. 52, 20246 Hamburg, Germany; 2 Department of Neurosurgery, University Medical Center Hamburg-Eppendorf, Hamburg, Germany

**Keywords:** Pituitary, Adenoma, PitNET, Synchronous, Methylation

## Case History

**Fig. 1:**
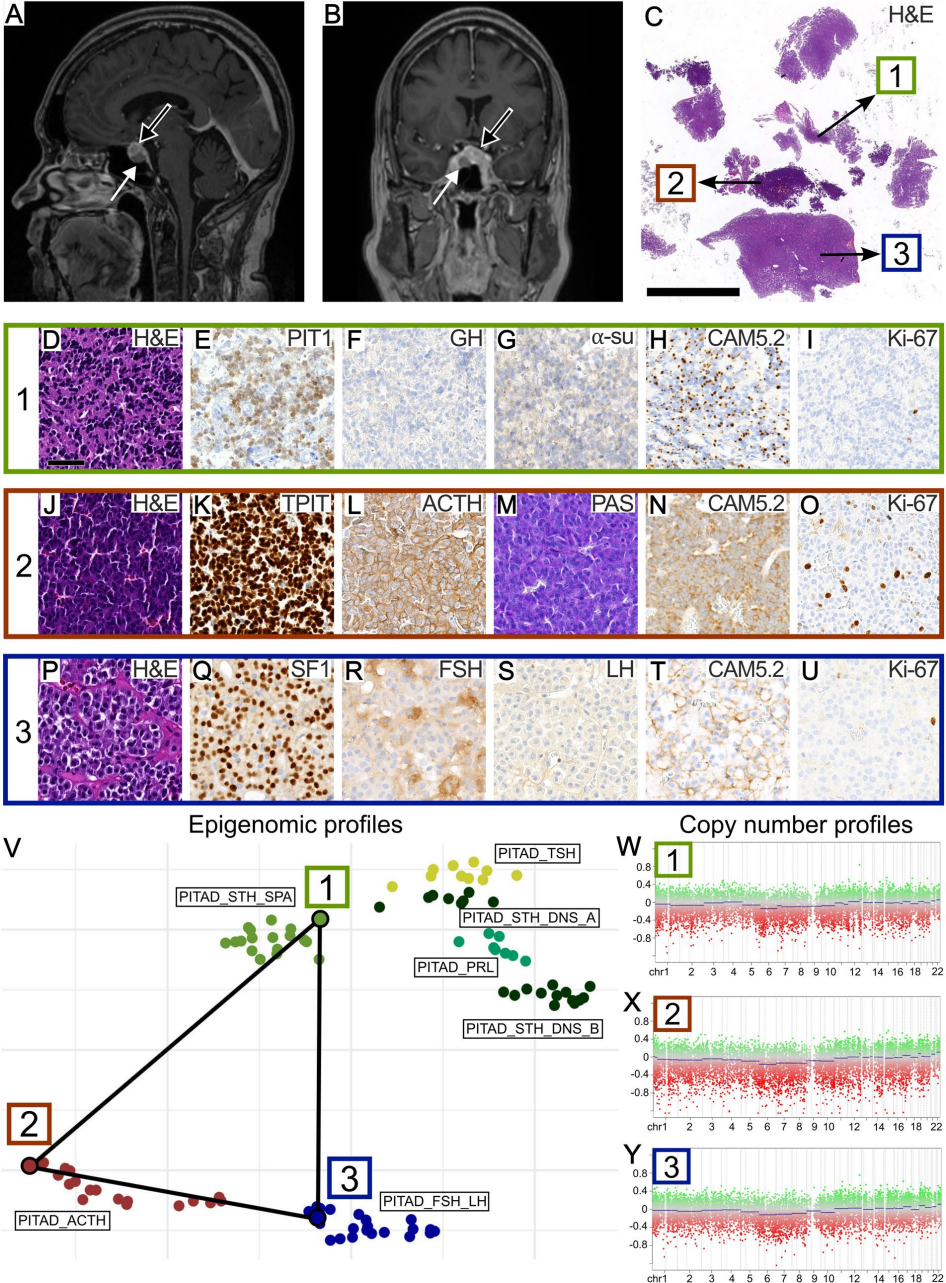
Radiological, histopathological, and molecular features of the pituitary tumors. **A**, **B** Contrast enhanced sagittal (**A**) and coronal (**B**) MR images showed a hypointense, well-defined intrasellar mass (white arrow) and an “enlarged pituitary gland” with inhomogeneous enhancement (black arrow). **C** Hematoxylin and eosin (H&E) staining revealed three distinct tumor components (labeled 1, 2, 3). Scale bar is 5 mm. **D–U** Histopathological images of the first (**D**–**I**), second (**J**–**O**) and third (**P**–**U**) tumor component are shown. Scale bar is 50 µm. **V** UMAP analysis based on the top 5000 variant CpG sites demonstrated epigenomic affiliation of the three tumor components 1, 2, and 3 with previously published reference samples of sparsely granulated somatotroph, corticotroph, and gonadotroph PitNETs [1], respectively. PITAD, pituitary adenoma; SPA, sparse; DNS, dense; STH, somatotropin (growth hormone (GH)); PRL, prolactin; TSH, thyroid stimulating hormone; ACTH, adrenocorticotropic hormone; FSH, follicle-stimulating hormone; LH, luteinizing hormone. **W**–**Y** Quiet copy number profiles were found in all three tumor components 1–3

A 56-year-old female was seen for imaging follow-up of a progressively enlarging pituitary mass, first identified on MRI 7 years ago. The initial imaging findings had raised suspicion of hypophysitis, particularly in the context of her prior treatment for malignant melanoma with immunotherapy. Within the right side of the sella, a well-demarcated hypointense component was discernable (Fig. 1A and B, white arrow). The rest of the pituitary appeared diffusely enlarged with inhomogeneous enhancement (Fig. 1A and B, black arrow). Laboratory tests revealed no signs of hypersecretion or pituitary insufficiency. In light of the progressive findings, the decision was made to proceed with transnasal transsphenoidal surgery. Gross complete resection of tumorous appearing tissue was achieved.

## What Is Your Diagnosis?

Histopathology revealed three distinct tumor components (Fig. 1C). The first component was characterized by scant infiltrates of deeply basophilic cells with barely discernible cytoplasm (Fig. 1D). These cells showed nuclear PIT1-immunopositivity (Fig. 1E) and immunonegativity for growth hormone (GH, Fig. 1F) and alpha-subunit (Fig. 1G). CAM5.2-immunostaining revealed fibrous bodies in roughly 80% of tumor cells (Fig. 1H). The Ki-67 proliferative index was less than 1% (Fig. 1I).

The second component consisted of densely packed, mildly pleomorphic cells with basophilic cytoplasm and round nuclei with dense chromatin (Fig. 1J). Strong nuclear TPIT expression (Fig. 1K), diffuse expression of ACTH (Fig. 1L), and strong PAS positivity (Fig. 1M) were present. CAM5.2 showed a diffuse staining pattern (Fig. 1N). The Ki-67 proliferative index was calculated at 3–5% (Fig. 1O).

The third component exhibited nests of monomorphic cells with chromophobic cytoplasm and round, chromatin-dense nuclei (Fig. 1P). These cells showed nuclear SF1 expression (Fig. 1Q), partially strong FSH expression (Fig. 1R), and no definite LH expression (Fig. 1S). CAM5.2 staining pattern was diffuse (Fig. 1T). The Ki-67 proliferative index was less than 1% (Fig. 1U).

Representative punch biopsies from each component were subjected to global DNA methylation profiling. Dimensionality reduction including previously published pituitary reference tumors [1], revealed epigenomic affiliation of the first, second and third components with sparsely granulated somatotroph, corticotroph and gonadotroph PitNETs, respectively (Fig. 1 V). For the first, second, and third component, the brain tumor methylation classifier (v12.8) calculated scores of 0.99, 0.99, and 0.79 for the “pituitary adenoma” methylation classes “subtype STH producing, subclass sparsely granulated”, “subtype ACTH producing”, and “subtype gonadotrophin producing”, respectively. All three tumors exhibited quiet copy number profiles (Fig. 1W–Y).

### Diagnosis

Three synchronous pituitary neuroendocrine tumors (PitNETs) of distinct lineages, comprising a sparsely granulated somatotroph, a densely granulated corticotroph, and a gonadotroph PitNET.

## Comment

Multiple synchronous PitNETs are rare and usually comprise two tumors of distinct lineages. Most patients exhibit signs of hormone excess due to the presence of at least one hormonally active tumor component [2]. The diagnosis of three synchronous PitNETs, each originating from one of the three distinct pituitary lineages, is exceptional, especially in a patient lacking both biochemical and clinical signs of hormone excess. Epigenomic analysis poses a powerful tool for molecular tumor classification, which confirmed the final diagnosis of this triple PitNET in line with previous epigenomic studies on pituitary neoplasms, including double PitNETs [1, 3]. Postoperatively, the patient recovered well showing no pituitary insufficiency, normal electrolyte levels, and no neurological deficit.

## Data Availability

The data processed in this study are available from the corresponding author upon reasonable request.
